# AmpC induction by imipenem in *Pseudomonas aeruginosa* occurs in the absence of OprD and impacts imipenem/relebactam susceptibility

**DOI:** 10.1128/spectrum.00142-24

**Published:** 2024-09-24

**Authors:** Shawn Freed, Jr, Nancy D. Hanson

**Affiliations:** 1 Department of Medical Microbiology and Immunology, Creighton University School of Medicine, Omaha, Nebraska, USA; 2 Creighton Center for Antimicrobial Resistance and Epidemiology, Creighton University School of Medicine, Omaha, Nebraska, USA; University at Albany, Albany, New York, USA

**Keywords:** *Pseudomonas aeruginosa*, imipenem/relebactam, OprD, AmpC

## Abstract

In the United States, carbapenem resistance in *Pseudomonas aeruginosa* is linked to the regulation of chromosomal resistance determinants, AmpC and OprD. The β-lactamase AmpC requires overexpression and genetic modifications to be capable of inhibiting imipenem activity. The outer membrane porin OprD can be downregulated or undergo genetic modifications that strongly correlate with imipenem non-susceptibility. Co-administration of imipenem and the β-lactamase inhibitor, relebactam, can lower imipenem minimal inhibitory concentrations and restore susceptibility. However, it is not understood how this occurs in *P. aeruginosa* isolates that do not overproduce AmpC or produce a functional OprD for imipenem entry. Therefore, we investigated whether imipenem could enter *P. aeruginosa* in the absence of OprD and whether any of the chromosomal β-lactamases (AmpC, OXA-50, and PIB-1) contributed to imipenem and/or imipenem/relebactam non-susceptibility. This investigation evaluated 17 imipenem non-susceptible clinical isolates and three laboratory strains of PAO1, two of which were porin transposon mutants for either *oprD* or *opdP*. Expression of OXA-50 and PIB-1 RNA was similar to PAO1. However, all 20 isolates exhibited *bla_ampC_* induction under sublethal exposure to imipenem. This occurred in the absence of detectable OprD protein in 18 isolates. Collectively, our data identify that (i) OprD was not the only channel required for imipenem entry and (ii) imipenem susceptibility can be restored by imipenem/relebactam due to the interaction between relebactam and *bla_ampC_* overexpression due to imipenem induction.

**IMPORTANCE:**

Infections caused by *Pseudomonas aeruginosa* are associated with high mortality and worsened clinical outcomes. The carbapenem, imipenem, has been combined with the β-lactamase inhibitor relebactam to treat carbapenem-resistant *P. aeruginosa*. Downregulation of the outer membrane porin, OprD is the major mechanism of imipenem resistance; however, relebactam inhibits the chromosomally encoded AmpC β-lactamase. We investigated how relebactam was able to reduce *P. aeruginosa* imipenem minimal inhibitory concentrations (MICs) in isolates in which OprD was downregulated. Our data show that imipenem is capable of entering the cell in the absence of OprD and capable of inducing the AmpC β-lactamase. The induction of AmpC provides a substrate for relebactam, impacting the imipenem MIC. The data presented support the use of an alternative porin(s) for entry of imipenem. This study provides the basis for further investigation into modifications of imipenem or similar molecules that would increase the affinity for other porins in isolates resistant to imipenem.

## INTRODUCTION

*Pseudomonas aeruginosa* harbors chromosomal mechanisms that render antibiotics from multiple classes ineffective (1). These mechanisms contribute to the high mortality and morbidity that is associated *with P. aeruginosa* infections (2). Carbapenem antibiotics have greater potency and higher stability against hydrolysis by the chromosomal β-lactamase produced by *P. aeruginosa*. However, a substantial amount of isolates still emerge resistant, with nearly 13% and 18% of *P. aeruginosa* infections resistant to at least one carbapenem in the United States and Europe, respectively (3, 4). Among carbapenem-resistant *P. aeruginosa*, only 2% are linked to the presence of a plasmid-encoded carbapenemase in the United States (5). Therefore, the majority of carbapenem-resistant strains of *P. aeruginosa* are the result of chromosomal mechanisms. Overexpression of the AmpC β-lactamase (*bla_ampC_*), efflux pump overexpression, and the downregulation of the OprD outer membrane porin are reported to have the biggest impact on carbapenem non-susceptibility (1, 6, 7).

Imipenem has been shown to evade overexpressed efflux mechanisms and remains a potent option against *P. aeruginosa* efflux mutants (8–10). However, *P. aeruginosa* may harbor variant chromosomal *bla_ampC_* alleles (also referred to as *Pseudomonas*-derived cephalosporinases or PDC alleles) with expanded spectrums of hydrolysis to certain cephalosporins and cephalosporin/β-lactamase combinations (7). Sublethal concentrations of imipenem can also induce the chromosomal *bla_ampC_*, which can lead to reduced susceptibility to imipenem (11). Relebactam was developed to help restore susceptibility to imipenem when given in combination. Relebactam preferentially binds to a wide range of *bla_ampC_* alleles, preventing them from hydrolyzing or binding to imipenem. Many *P. aeruginosa* isolates that are non-susceptible to imipenem alone, have been shown to be susceptible when paired with relebactam (12).

OprD is an outer membrane channel that selectively allows small peptides and basic amino acids to permeate into the periplasmic space (13). It has been shown that imipenem translocates through OprD (14). Modifications in the *oprD* gene can lead to a loss in porin production or confirmational changes to the structure of the porin, both of which can lead to imipenem non-susceptibility (15, 16). OprD has 17 related homologues, 8 of which have substrates experimentally determined (17). Among these is the porin, OpdP. Genetic analysis revealed OpdP to be the most similar to OprD and *in silico* studies have implicated that imipenem can be translocated through OpdP (18, 19).

It is assumed that the major mechanism of resistance to imipenem in most clinical isolates is due to the absence of a functional OprD. The question is, why does relebactam have an effect on imipenem minimal inhibitory concentrations (MICs) in the absence of a functional OprD? We hypothesized that imipenem gains access into OprD mutant cells and induces *bla_ampC_* allowing a substrate for relebactam binding, thus lowering the imipenem MIC. To test this hypothesis, we investigated whether *bla_ampC_* induction occurred in the absence of a functional OprD in both clinical isolates and an OprD transposon mutant of the laboratory strain, PAO1.

The data presented in this study demonstrates three main points: (i) In the absence of OprD, *bla_ampC_* expression can be induced in the presence of imipenem, (ii) OprD is not the only entry point for imipenem, and (iii) overproduction of AmpC can contribute to imipenem non-susceptibility, as has been noted by others (11).

## RESULTS

The differences seen in *P. aeruginosa* MICs between treatment of imipenem alone versus imipenem/relebactam infer that increased levels of AmpC production can contribute to the susceptibility of imipenem/relebactam (Table 1). We hypothesized that in order for relebactam to have an impact on the imipenem MIC, the isolate must express *bla_ampC_* at low levels and not high levels as those seen in a derepressed mutant. In this scenario, the basal level *bla_ampC_* expression is induced upon treatment with imipenem thus allowing relebactam as a substrate for binding.

**TABLE 1:** Imipenem MICs and RNA transcript levels of resistance mechanisms for *Pseudomonas aeruginosa* clinical isolates and lab strains

	MIC in µg/mL (susceptibility phenotype)^*b*^		RNA fold-change relative to PAO1 (coefficient of variance)^*c*^				
Strain^*a*^	Imi	Imi/Rel	AmpC	OprD	OpdP	Oxa-50	Pib-1
PAO1	0.25 (S)	0.5 (S)	1 (0.04)	1 (0.01)	1 (0.04)	1 (0.02)	1 (0.02)
1203673	4 (I)	0.12 (S)	7 (0.04)	−1742 (0.03)	1 (0.04)	1 (0.02)	−1 (0.03)
1373052	4 (I)	0.5 (S)	2 (0.02)	−340 (0.03)	14 (0.01)	−11 (0.02)	−6 (0.03)
1328797	4 (I)	2 (S)	2 (0.04)	−2 (0.04)	8 (0.06)	−1 (0.03)	−2 (0.07)
1499564	8 (R)	0.25 (S)	5 (0.02)	4 (0.04)	6 (0.06)	1 (0.02)	−11 (0.03)
1352384	8 (R)	1 (S)	3 (0.04)	−31,350 (0.00)	4 (0.01)	−1 (0.02)	−7 (0.02)
1234087	8 (R)	8 (R)	3 (0.02)	−4 (0.03)	6 (0.04)	3 (0.03)	−5 (0.02)
1378361	16 (R)	0.25 (S)	6 (0.02)	ND	ND	2 (0.01)	−4 (0.03)
1219235	16 (R)	2 (S)	6 (0.04)	−64,092 (0.03)	2 (0.02)	2 (0.05)	−4 (0.06)
1214215	16 (R)	16 (R)	26 (0.03)	1 (0.05)	10 (0.07)	1 (0.03)	−5 (0.04)
1217799	32 (R)	0.5 (S)	2 (0.03)	−28 (0.04)	20 (0.03)	−2 (0.03)	−7 (0.04)
1485138	32 (R)	4 (I)	8 (0.02)	−18,814 (0.03)	12 (0.05)	2 (0.02)	−4 (0.05)
720171	32 (R)	32 (R)	2 (0.02)	−106 (0.05)	22 (0.05)	2 (0.03)	−9 (0.06)
726579	64 (R)	8 (R)	1 (0.01)	−24,146 (0.04)	269 (0.01)	−2 (0.03)	−5 (0.05)
763834	64 (R)	16 (R)	296 (0.01)	−4 (0.00)	4 (0.05)	−2 (0.01)	−5 (0.01)
717609	64 (R)	32 (R)	2 (0.01)	−114 (0.01)	6 (0.04)	1 (0.02)	−2 (0.06)
750050	128 (R)	>32 (R)	3 (0.02)	ND	123 (0.02)	−4 (0.01)	−1 (0.08)
787256	256 (R)	>32 (R)	4 (0.03)	ND	3 (0.04)	2 (0.01)	−3 (0.03)
PW2742	32 (R)	4 (I)					
PW8575	2 (S)	0.5 (S)					
PW7954	0.5 (S)	0.5 (S)					
OprDKOCR	64 (R)	2 (S)					

^*a*^ Strains PW2742 (*oprD*-E12::ISphoA/hah), PW8575 (*opdP*-H11::ISphoA/hah) and PW7954 (*ampC*-D09::ISphoA/hah) obtained from Manoil Transposon Library (20). Strain OprDKOCR was obtained from Creative Biogene. Clinical isolates were obtained from Merck.

^*b*^ Susceptibilities of clinical isolates were supplied by IHMA, Inc. and confirmed locally via E-test. Lab strain susceptibilities obtained via E-test. Phenotypic interpretation: S, susceptible; I, intermediate; R, resistant. Imi, imipenem; Imi/Rel, imipenem-relebactam.

^*c*^ Relative RNA transcript levels of three chromosomal β-lactamase enzymes (*ampC*, *oxa-50*, and *pib-1*) and two porins (*oprD* and *opdP*) determined using qRT-PCR and fold-change calculated using 2^−ΔΔCT^ method with PAO1 as the comparator. Fold-change values rounded to the nearest whole number. Coefficient of variance included in parenthesis. ND, not detected after 40 amplification cycles.

Therefore, our first step was to evaluate *bla_ampC_* transcript levels in imipenem non-susceptible clinical isolates using qRT-PCR. The non-susceptible imipenem/relebactam MICs for isolates evaluated in this study ranged from 4 to 256 µg/mL (Table 1). In the majority of the isolates evaluated the *bla_ampC_* expression was low ranging from two- to sevenfold differences in expression when compared to PAO1. Seven of 17 isolates were susceptible to imipenem/relebactam, while all of the isolates were non-susceptible to imipenem alone. One isolate (1485138) remained intermediate to imipenem/relebactam and expressed *bla_ampC_* eightfold higher than PAO1. The two isolates that expressed *bla_ampC_* RNA at levels of 26- and 296-fold higher than PAO1 were considered derepressed mutants and remained imipenem resistant in the presence of imipenem/relebactam. Six isolates had *bla_ampC_* expression levels of only two- to fourfold higher than PAO1 but our hypothesis that the basal level of *bla_ampC_* expression would correlate with susceptibility to imipenem/relebactam did not hold for these isolates. However, *P. aeruginosa* encodes two additional chromosomally encoded β-lactamases in addition to *bla_ampC_*: *bla_oxa-50_* and *bla_pib-1_*. The transcript levels of those enzymes could explain the lack of susceptibility to those six strains resistant to imipenem/relebactam. However, the transcript levels of these chromosomal genes were low. *bla_oxa-50_* expression ranged from a 3-fold increase to an 11-fold decrease and *bla_pib-1_* ranged from no change to an 11-fold decrease when compared to PAO1 (Table 1).

**Fig 1:**
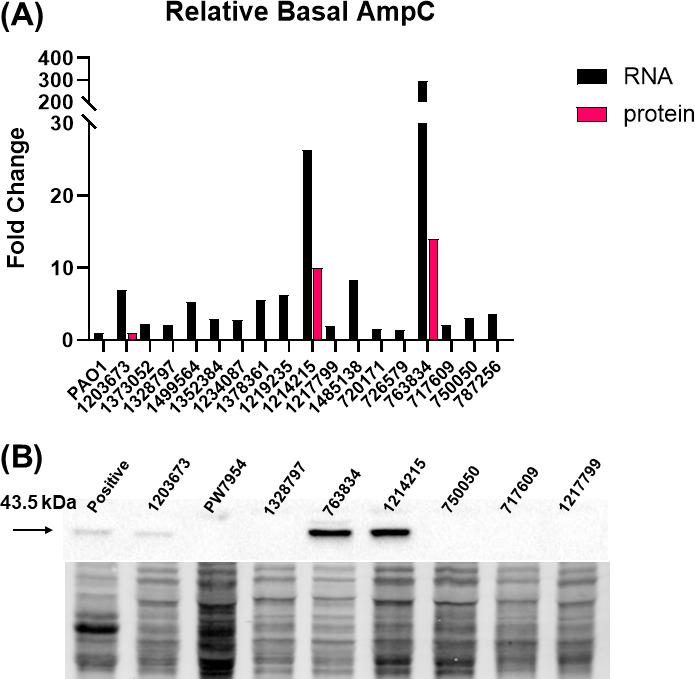
Clinical isolates exhibit increased RNA transcript levels of *ampC*, but protein levels remain below limit of detection. (**A**) Cells were grown to mid-logarithmic phase before being harvested for qRT-PCR (black bars) and western blot (magenta bars) analysis. Fold-change in RNA transcript was calculated using 2^−ΔΔCT^ method using PAO1 as the comparator isolate. All analyses were conducted in triplicate, using fresh cultures each time. Coefficient of variance was <10%. (**B**) Representative western blot showing distinct bands for 1203673, 1214215, and 763834. Total protein image is used as a loading control and normalization factor. All experiments were conducted in triplicate, using fresh cultures each time. PAO1 AmpC protein was below the limit of detection, an alternate isolate was used as a positive control and PW7954 (*ampC*-D09::ISphoA/hah) was used as a negative control. Densitometry analysis from western blot was used to calculate fold-change using 1203673 as the comparator isolate.

Basal levels of *bla_ampC_*expression were so low that enzymatic activity would be hard to detect due to a low level of protein production (21, 22). To show that in the absence of imipenem induction, AmpC protein levels are low compared to isolates with derepressed transcript levels we evaluated western blots to determine the relative protein levels of AmpC in the clinical isolates compared to PAO1 (Fig. 1B). Only 3 of the 17 isolates (17.6%): 1203673, 1214215, and 763834, had detectable levels of AmpC protein. Isolates 1214215 and 763834 exhibited the highest transcript levels of 26- and 296-fold, respectively, compared to PAO1 and the highest level of protein production. Both isolates were resistant to imipenem and imipenem/relebactam (Fig. 1A). In contrast, 1203673 which was intermediate (MIC = 4) to imipenem alone and susceptible to imipenem/relebactam had only a sevenfold increase in *bla_ampC_* expression relative to PAO1 (Fig. 1A). Interestingly, 4 of the 17 isolates (23.5%), 1499564, 1378361, 1219235, and 1485138, had transcript levels between five- and eightfold higher than PAO1 but no detectable AmpC protein in the absence of imipenem was observed (Fig. 1A and B). Three of those isolates (1499564, 1214215, and 1219235) were susceptible to imipenem/relebactam (MIC = 0.25–2 µg/mL) (Table 1). In total, only 3 of the 17 clinical isolates had detectable AmpC protein in the absence of induction by imipenem. Seven of the 17 were non-susceptible to imipenem/relebactam even though AmpC was only detectable by RNA analysis and not protein. These data showed that even though *bla_ampC_* RNA was detected, protein synthesis was below the level of detection using western analysis and correlated with imipenem/relebactam susceptible isolates. Six isolates had no detectable AmpC protein in the absence of imipenem but were resistant to imipenem/relebactam indicating other mechanisms were involved in the resistant phenotype.

It is possible that modifications in genes associated with *bla_ampC_* expression or modifications in the *ampC* structural gene could have impacted the expression and therefore the imipenem/relebactam non-susceptibility.

It has been suggested that chromosomal *bla_ampC_* can influence the imipenem MIC through overproduction of the enzyme in conjunction with hydrolytic properties associated with allelic variants of the enzyme (23–26). However, *bla_ampC_* alleles containing an alanine substitution at residue 105 in addition to the overexpression of the enzyme have not been shown to contribute to imipenem non-susceptibility, but have been shown to impact the newer β-lactam/β-lactamase inhibitor combinations and antipseudomonal cephalosporins (6, 7). Nonetheless, we used whole-genome sequencing (WGS) to identify the *bla_ampC_* allele for each clinical isolate and any mutations in genes associated with the induction pathway for *bla_ampC_*, including mutations within *ampD*, *ampR*, *nagZ*, *dacB* (PBP-4), and *sltB1* (27). Nine different *bla_ampC_* (PDC) variants were identified in the 17 isolates with PDC-3 present in 7 of the 17 isolates. *bla_ampC_* alleles encoding the T105A substitution were present in 15 of the 17 of those isolates with isolates 1328797 and 1378361 having a wild-type codon at position 105 (Table 2). Although there were a few mutations in genes associated with the induction pathway only two of the isolates (1214215; PDC-3 and 763384; PDC-24) overexpressed *bla_ampC_* at both the RNA and protein levels compared to PAO1. These two isolates were resistant to imipenem and resistance was not alleviated by the addition of relebactam. However, all isolates except 1203673, 1373052, and 1234087 had mutations in *ampD* and 1373052, 1328797, 1219235, 1485138, and 717609 had mutations within *ampR* and yet none of these isolates had increased basal levels of *bla_ampC_* (Table 2).

**TABLE 2:** Molecular characterization of resistance mechanisms and induction pathways in clinical isolates of *Pseudomonas aeruginosa*

Strains^*a*^	OprD^*e*^	*ampC^c^*	ampD	ampR	nagZ	*sltB1^d^*	PBP4
PAO1^*b*^	WT	PDC-1	WT	WT	WT	WT	WT
1203673	9 AA	PDC-59	WT	3 AA	WT	WT	WT
1373052	9 AA	PDC-34	WT	3 AA	WT	WT	1 AA
1328797	Stop codon	PDC-98	2 AA	WT	WT	WT	WT
1499564	3 AA	PDC-3	2 AA	WT	WT	WT	WT
1352384	Stop codon	PDC-5	1 AA	WT	WT	WT	1 AA
1234087	26 AA	PDC-8	WT	WT	WT	WT	1 AA
1378361	72 AA	PDC-1	3 AA	WT	WT	WT	WT
1219235	Stop codon	PDC-3	1 AA	8 AA, one deletion	WT	WT	WT
1214215	68 AA	PDC-3	3 AA	WT	WT	1 AA	WT
1217799	Stop codon	PDC-8	1 AA	WT	WT	WT	WT
1485138^*b*^	89 AA	PDC-3	1 AA	8 AA, one deletion	WT	*	WT
720171^*b*^	IS element	PDC-3	1 AA	WT	WT	*	1 AA
726579	Stop codon	PDC-16	1 AA	WT	WT	WT	WT
763834	25 AA, two deletions	PDC-24	4 AA	WT	WT	WT	1 AA
717609^*b*^	Stop codon	PDC-3	2 AA	8 AA, one deletion	WT	*	WT
750050	Stop codon	PDC-5	3 AA	WT	1 AA	1 AA	1 AA
787256	Stop codon	PDC-3	1 AA	WT	WT	WT	1 AA

^*a*^ Isolates were sequenced using Illumina MiSeq unless noted otherwise.

^*b*^ Isolates sequenced by SeqCenter.

^*c*^ PDC alleles provided by Merck.

^*d*^ Three sequences could not be retrieved from SeqCenter analysis, denoted by *.

^*e*^ WT, wild-type; AA, amino acid modifications.

**Fig 2:**
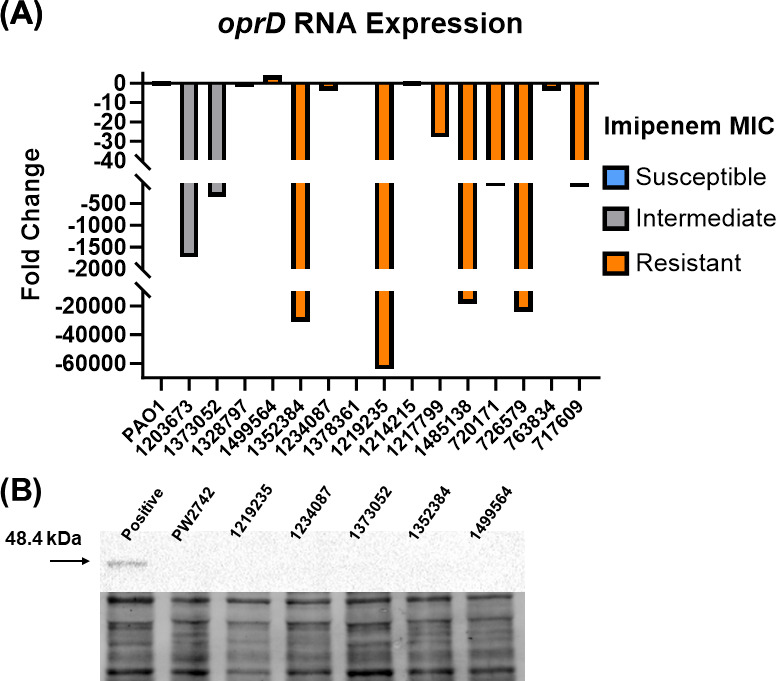
Decreased *oprD* transcript levels and protein production correlates with non-susceptibility to imipenem. (**A**) Cells were grown to mid-logarithmic phase before being harvested for qRT-PCR and fold-change was calculated using 2^−ΔΔCT^ method using PAO1 as the comparator isolate. Imipenem susceptibility phenotypes are indicated by color (gray = intermediate, orange = resistant). Three isolates (1378361, 750050, and 787256) exhibited no amplification after 40 cycles. Coefficient of variance was ≤5%. (**B**) Cells grown as stated above. Representative western blot showing lack of protein detection for all clinical isolates. Total protein image is used as a loading control and normalization factor. Densitometry analysis was not performed. All experiments were conducted in triplicate, using fresh cultures each time. PAO1 was used as a positive control and PW2742 (*oprD*-E12::ISphoA/hah) was used as a negative control.

Loss of OprD plays a critical role in the emergence of imipenem non-susceptibility (13–15, 24, 26). Therefore, we wanted to examine the contribution of OprD to the various susceptibility patterns observed in these isolates by evaluating the RNA and protein production of OprD. All the clinical isolates had reduced *oprD* transcript levels compared to PAO1, with three isolates (1378361, 750050, and 787256) having no detectable transcript (Fig. 2A). Five clinical isolates (1328797, 1499564, 1234087, 1214215, and 763834) had between no change and a fourfold decrease relative to PAO1 but the imipenem MICs for those isolates ranged from 4 to 64 µg/mL (Fig. 2A; Table 1). Although the RNA levels for *oprD* varied, none of the isolates had detectable levels of OprD protein using western blot analysis (Fig. 2B). This was expected for the 12 isolates (70.6%) that showed large negative fold-changes in transcript levels, but surprising for the five strains (1328797, 1499564, 1234087, 1214215, and 763834) with *oprD* transcript levels similar to PAO1 (Fig. 2B). The lab strains, PAO1 and *opdP*-H11::IsphoA/hah had detectable OprD protein bands while *oprD*-E12::IsphoA/hah did not, as expected (Fig. 2B; Fig. S1A).

Our inability to detect OprD protein prompted us to analyze the sequences for mutations, either within the epitope used to generate the polyclonal antibody or mutations that would truncate the protein. Every isolate harbored amino acid changes compared to PAO1. Nine isolates (52.9%) had a premature stop codon or an IS element that truncated the sequence (Table 2). The remaining isolates were divided into two groups: three isolates (17.6%) with less than 10 mutations (3–9 AA) and five isolates (29.4%) with greater than 10 mutations (25–89 AA) (Table 2). Isolates with less than 10 amino acid changes had lower imipenem and imipenem/relebactam MICs compared to those with greater than 10 alterations or those with premature stop codons, despite undetectable OprD protein and varying transcript levels (Tables 1 and 2). However, despite most isolates (82.4%) having 25 or more amino acid changes or truncations in OprD, relebactam was still able to lower the imipenem/relebactam MICs in 11 of the 17 (64.7%) isolates. These data suggested imipenem entered the cell and that relebactam acted upon its target, AmpC.

### AmpC induction by imipenem occurs in absence of OprD

**Fig 3:**
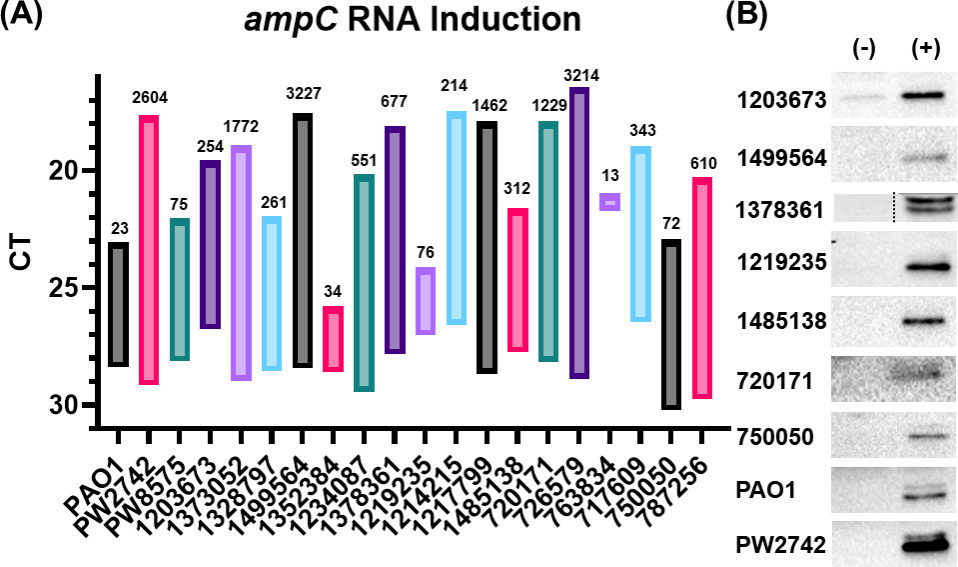
Induction of *ampC* RNA and protein occurs upon imipenem treatment. (**A**) Isolates were grown to mid-logarithmic phase then treated with 1/4^th^ their respective imipenem MIC for 15 minutes and subsequently harvested for qRT-PCR and fold-change was calculated using 2^-ΔΔCT^ method using untreated respective samples as a Comparator. The lower bound of the column represents the basal cycle threshold (CT) for *ampC* and the upper bound is the induced CT. The data label indicates the fold-change in transcript levels. Coefficient of variance was <10%. (**B**) Western blot samples were harvested as above. Representative blots show induction of protein following treatment (‘-’ untreated, ‘+’ treated). All experiments were conducted in triplicate, using fresh cultures each time.

Although the majority of the isolates did not produce detectable levels of AmpC using western blot analysis, treatment of the isolates with imipenem/relebactam lowered the imipenem MICs for 11 of the 17 (64.7%) clinical isolates tested (Table 1). Therefore, we investigated whether imipenem would induce *bla_ampC_* transcript levels and protein production in the clinical isolates. Induction of *bla_ampC_* occurred for all isolates tested (Fig. 3A). RNA induction among the isolates varied dramatically, but this was due, in part, to the basal level of *bla_ampC_* expression for each isolate. Six isolates (1373052, 1499564, 720171, 726579, 717609, and 787256) had greater than 1,000-fold increase in RNA expression compared to the respective uninduced level. This large increase in expression in the presence of imipenem correlated with a low *bla_ampC_* basal level of expression with basal level CTs ≥28, (Fig. 3A). Isolates 1203673, 1214215, and 763834 had higher levels of basal *bla_ampC_* transcript (CTs of 26 and 21) and detectable protein but, still exhibited *bla_ampC_* induction by imipenem of 254-, 214-, and 13-fold, respectively (Fig. 3A). Isolates 1352384 and 750050 showed very little induction of RNA, 34- and 16-fold, respectively, despite having low basal levels (CTs 28–30) (Fig. 3A). Isolates that had *oprD* transcript levels similar to PAO1 (1499564, 1234673, 1214215, and 763834) showed a wide range of *bla_ampC_* RNA induction of between 13- and 3,227-fold (Table 1; Fig. 3A). The variance in *bla_ampC_* induction was similar to isolates that did not express high levels of *oprD* or had premature stop codons with increases between 34- and 3,214-fold (Table 1; Fig. 3A). These variations observed in the induction potential suggested modifications within the known genes of the induction pathway. Therefore, sequence data were obtained for all isolates (Table 2). Isolate 1352384 had no unique mutations within *ampR*, *ampD*, or *dacB* (PBP-4) (Table 2). *dacB* did harbor an A394P mutation in 1352384, but this residue was also shared with four other isolates showing high levels of *bla_ampC_* RNA induction, including the derepressed mutant 763834 (Fig. 3A). Isolate 750050 harbored a unique A107S mutation within *nagZ* and a unique D283E mutation within the sequence for the lytic transglycosylase, SltB1 (Table 2). These mutations could contribute to the lower *bla_ampC_* induction potential (16-fold) observed. The lab strains: PAO1, *oprD*-E12::ISphoA/hah, and *opdP*-H11::ISphoA/hah, were also examined and showed levels of *bla_ampC_* RNA induction that ranged from 42 to 1,600-fold (Fig. 3A). All 17 clinical isolates and the three lab strains had detectable AmpC using western analysis following imipenem induction (Fig. 3B).

These data, taken together, suggest that imipenem entered the cell in the absence of OprD, which induced AmpC production providing the substrate for relebactam. (Table 1; Fig. 2B and 3B).

### OpdP may contribute to imipenem/relebactam uptake, but is not essential

**Fig 4:**
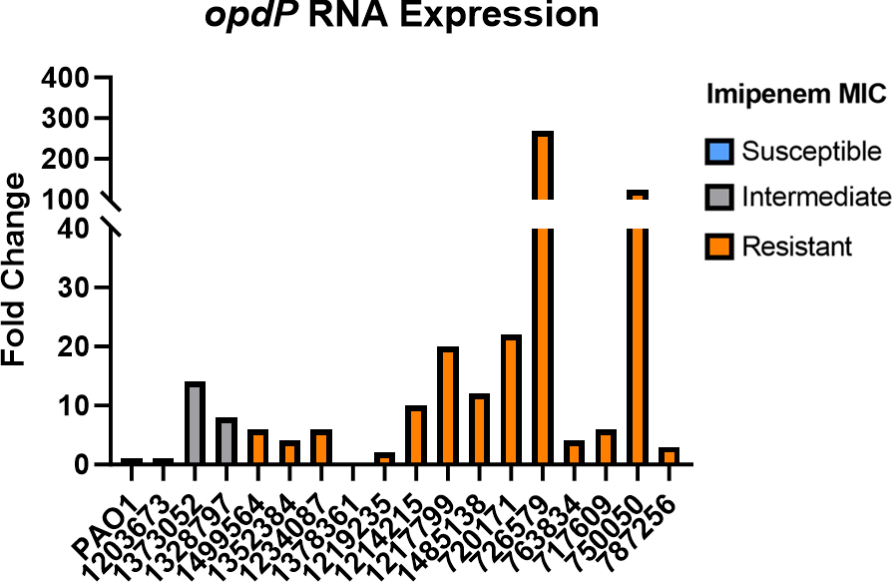
Clinical isolates exhibit *opdP* transcript at varying levels. Cells were grown to mid-logarithmic phase before being harvested for qRT-PCR and fold-change was calculated using 2^−ΔΔCT^ method using PAO1 as the comparator isolate. Imipenem susceptibility phenotypes are indicated by color (gray = intermediate, orange = resistant). 1378361 exhibited no amplification after 40 cycles. All experiments were conducted in triplicate, using fresh cultures each time. Coefficient of variance was ≤7%.

Previously published studies have suggested an alternate porin within the OprD (OccD) family, OpdP, may be capable of translocating imipenem into the periplasmic space of *P. aeruginosa* (19, 28, 29). OpdP production may compensate for the loss of functionality in OprD seen frequently in *P. aeruginosa* clinical isolates. We investigated whether the clinical isolates in this study had increased *opdP* transcript levels compared to PAO1. Expression of *opdP* was observed in 16 (94.1%) clinical isolates with isolate 1378361 being the only exception (Fig. 4). Of these, 15 (88.2%) isolates had increased relative *opdP* expression compared to PAO1, while isolate 1203673 showed the same level as PAO1 (Fig. 4). Despite this, all 16 isolates had CT values for *opdP* (36–28), suggesting that OpdP was being produced albeit at low levels in these organisms in the absence of OprD. Therefore, OpdP may provide a point of entry for imipenem which could explain the imipenem/relebactam MICs observed. However, isolate 1378361 did not show any transcript for *opdP* nor *oprD*, yet surprisingly *bla_ampC_* was inducible in the presence of imipenem (Fig. 3 and 4). It is possible, but unlikely, that imipenem was capable of passively diffusing through the outer membrane. Rifampicin has been used as an indicator for membrane integrity for *P. aeruginosa* (30, 31). Therefore, rifampicin disk diffusion tests were performed (Fig. S5). These data indicated that membrane integrity was not compromised, and passive diffusion was unlikely. These data imply that another porin(s), in the absence of OprD and/or OpdP, was also capable of allowing imipenem into the cell.

### RNAseq analysis of isolates under imipenem challenge

**Fig 5:**
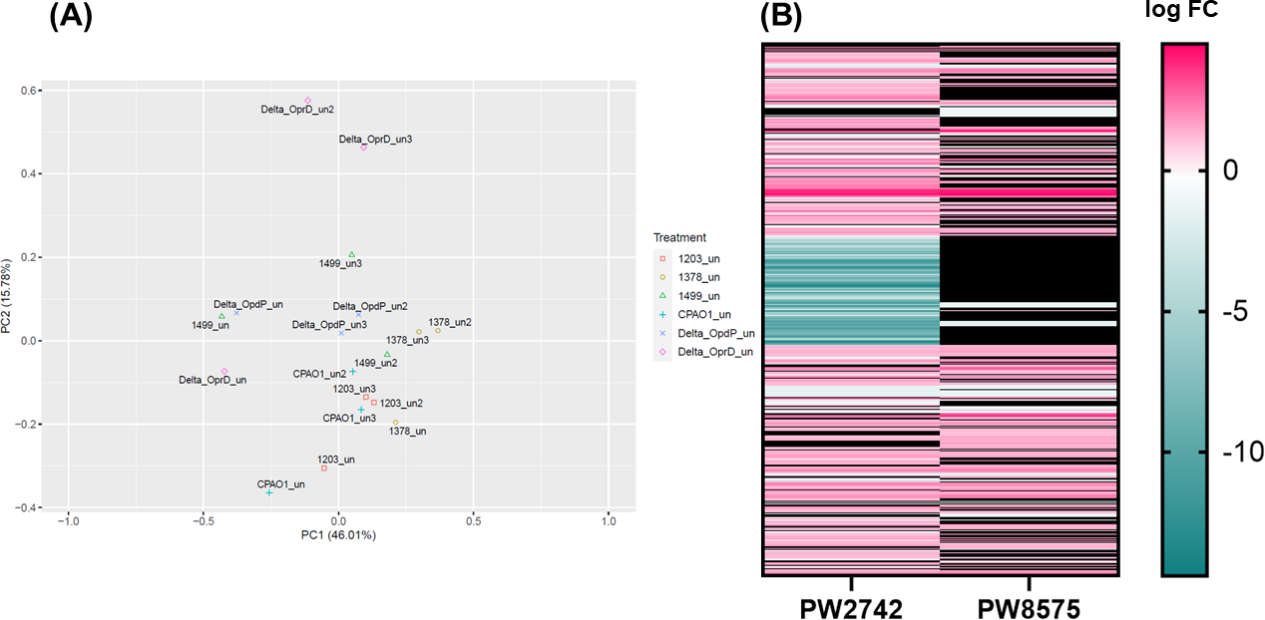
Transcriptomic analysis of three *Pseudomonas aeruginosa* laboratory strains and three clinical isolates in the absence of imipenem. (**A**) Principal component analysis considering all quantified genes. Each isolate is represented by a different symbol and color (see key). Biological replicates are labeled* within the graph with the replicate number and designated as “uninduced.” ΔOprD and ΔOpdP replicates refer to the transposon mutants *oprD*-E12::ISphoA/hah and *opdP*-H11::ISphoA/hah, respectively. (**B**) Heatmap showing differentially expressed genes of the PW2742 (*oprD*-E12::ISphoA/hah) and PW8575 (*opdP*-H11::ISphoA/hah) transposon mutants compared to PAO1. Log fold-change (FC) is indicated by the colors shown in the key. Blacked out genes are not significantly differentially expressed from PAO1 (*P* < 0.05, log FC > │1│). All experiments were conducted in triplicate, using fresh cultures each time. *A labeling error for ΔOprD_un2 was corrected during analysis.

We hypothesized that imipenem induction of *bla_ampC_* when combined with relebactam would provide a target for relebactam resulting in a decrease in imipenem/relebactam MICs. However, some isolates in which *bla_ampC_* was inducible in the presence of imipenem did not show a decrease in imipenem/relebactam MICs. These data suggested that mechanisms other than *bla_ampC_* or any of the *bla_ampC_* alleles identified might be involved in imipenem/relebactam resistance (Tables 1 and 2). To understand what cellular modifications occurred when cells were treated with imipenem, we evaluated the transcriptome of the three lab strains: PAO1, *oprD*-E12::ISphoA/hah, and *opdP*-H11::ISphoA/hah, and three clinical isolates selected for their unique phenotypes. Isolate 1203673 had detectable AmpC protein, downregulated *oprD* transcript (1,742-fold decrease), and an imipenem MIC of 4 µg/mL. Isolate 1378361 did not express *oprD* or *opdP*, yet *bla_ampC_* was still inducible by imipenem indicating another porin entry site. Isolate 1499564 encoded the *bla_ampC_* allele PDC-3 and was resistant to imipenem alone (8 µg/mL) but susceptible to imipenem/relebactam (0.25 µg/mL) as well as ceftazidime, cefepime, and pipercillin (data not shown). This isolate also had similar transcript levels of both *bla_ampC_* and *oprD* compared to PAO1. Principal component analysis (PCA) of the biological replicates for transcription in the absence of imipenem was performed on the six isolates (Fig. 5A). Evaluation of 5,853 genes showed that the transposon mutants deficient in one of the two porins clustered more closely with clinical isolates than with WT PAO1 (Fig. 5A). The clinical isolate 1203673 clustered closely with PAO1, which was not surprising given several genotypic similarities in transcript levels as well as imipenem MICs (Table 1). Replicates of transcript from isolate 1499564 clustered the furthest from each other, while *oprD*-E12::ISphoA/hah replicate isolates clustered the furthest from any of the isolates. Further analysis of the transcriptome related to PAO1 was carried out (Fig. 5B).

Comparative analysis between *oprD*-E12::ISphoA/hah and PAO1 revealed a large contiguous set of 145 genes (*czcC*/PA2522 – PA2377) deleted from the *oprD*-E12::ISphoA/hah genome. (Fig. 5B; Supplemental File 1). Many of these genes were hypothetical, but interestingly porins *opdT* and *opdJ* were deleted. Coupled with *oprD*, this isolate was deficient in three OprD-related porins but was capable of AmpC induction when treated with imipenem. In light of this, a CRISPR KO of *oprD* was obtained and imipenem induction experiments and AST was performed and validated the results observed in the transposon mutant.

**Fig 6:**
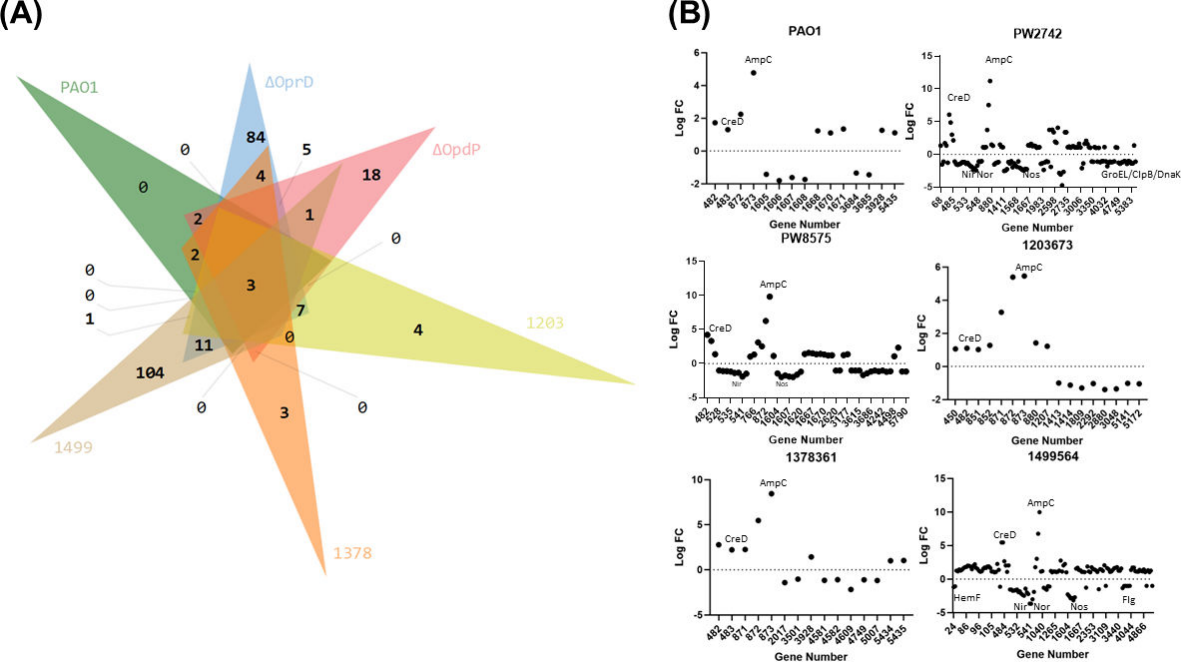
Transcriptomic analysis of *Pseudomonas aeruginosa* isolates treated with sublethal concentrations of imipenem. (**A**) Venn diagram showing significantly (*P* < 0.05) differentially expressed genes that meet a threshold of log fold-change (FC) > │1│Generated using InteractiVenn (32). Comparative analysis for lab strains: PAO1, ΔOprD (PW2742 or *oprD*-E12::ISphoA/hah), and ΔOpdP (PW8575 or *opdP*-H11::ISphoA/hah), and clinical isolates: 1203673, 1378361, and 1499564. (**B**) Differentially expressed genes under imipenem challenge for the isolates PAO1, PW2742, PW8575, 1203673, 1378361, and 1499564. 1203 = 1203673, 1378 = 1378361, 1499 = 1499564. *Pseudomonas* Genome Database gene annotation numbers are used (33). *creD, ampC, groEL/dnaK/clpB* indicate the position of the respective gene within the differentially expressed sample set. Nir, Nor, Nos, Flg, and HemF indicate the position of the respective operons within the differentially expressed sample set. All experiments were conducted in triplicate, using fresh cultures each time.

Differential transcript expression was determined for six isolates (PAO1, *oprD*-E12::IsphoA/hah, *opdP*-H11::IsphoA/hah, 1203673, 1378361, and 1499564) in the presence and absence of imipenem (Fig. 6A and B). Significance for gene enrichment (increased relative transcript) or depletion (decreased relative transcript) was based on the thresholds *P* < 0.05 and log FC > │1│. Among all isolates, comparable increases in *bla_ampC_* transcript in the presence of imipenem were observed when the RNAseq data were compared to the RT-qPCR data (Table 3). PAO1 and isolate 1378361 (did not express either *oprD* or *opdP)* (Table 1) showed the fewest changes in the presence of imipenem, with each isolate having 15 differentially expressed genes (DEGs). Of those 15 DEGs in the presence of imipenem, there were no unique genes compared to the other five isolates for PAO1 (Fig. 6A). In the presence of imipenem, three of the DEGs in isolate 1378361 were uniquely differentially expressed compared to the other five isolates examined (Fig. 6A). Isolate 1203673 had 4 of the 17 significant DEGs that were unique, while isolate 1499564, which had increased transcript levels of both *oprD* and *opdP* compared to PAO1, had 104 of the 142 DEGs that were unique (Fig. 6A). Both transposon mutants were analyzed when treated with imipenem: *opdP*-H11::IsphoA/hah had 18 of the 52 DEGs unique genes, whereas *oprD*-E12::IsphoA/hah had 84 of the 139 DEGs that were unique compared to the other five isolates. Among all six isolates, only three DEGs were shared: *creD*, *ampC*, and the hypothetical protein: PA4112, and all three were enriched in the presence of imipenem (Fig. 6A and B).

**TABLE 3:** Expression of *ampC* and differentially expressed genes from transcriptomic analysis^*a*^

	PAO1		PW2742		PW8575		1203673		1378361		1499564	
	RT-qPCR	RNAseq	RT-qPCR	RNAseq	RT-qPCR	RNAseq	RT-qPCR	RNAseq	RT-qPCR	RNAseq	RT-qPCR	RNAseq
ampC	23	27.25	2,604	2,359.49	75.00	911.91	254.00	43.95	677.00	346.94	3,227.00	1,008.92
aroE	−1	ns	1	ns	−1	ns	−1	ns	−1	ns	−1	−2.28
creD	1	3.30	15	67.22	2	18.77	1	2.14	3	6.88	10	43.82
groEL	−2	ns	−2	−2.25	−3	ns	−2	ns	−2	−2.24	−2	ns
dnaK	−2	ns	−1	−2.11	−4	ns	−4	ns	−2	−2.26	1	ns
clpB	−2	ns	−1	−2.39	−3	ns	−3	ns	−2	−2.16	1	ns
nirN	−2	ns	−2	−2.81	−3	−2.02	−2	ns	−2	ns	−4	−2.95
nor	−3	ns	−7	−5.28	−6	−2.78	−4	ns	−2	ns	−45	−8.08
nosL	−3	−2.68	−5	−4.50	−5	−3.94	−5	ns	−2	ns	−5	−5.82

^*a*^ Fold-change in RNA transcript level was determined using isolates induced by sublethal concentrations of imipenem and comparing them to isolates left untreated. RT-qPCR analysis conducted on *ampC* and various operons found either enriched or depleted transcriptomic analysis. Fold-change for both data sets were calculated using 2^−ΔΔCT^ method. ns, not significant

It was interesting to note, that between all the isolates, RNA transcripts of the operons for nitric oxide, nitrite, and nitrate reduction were commonly depleted. Nitric oxide reductase and nitrite reductase operons were depleted between log FC −2 and log FC −4 in 1499564 and *oprD*-E12::IsphoA/hah. (Fig. 6B). A nitrous oxide operon was found to be depleted in 1499564, *oprD*-E12::IsphoA/hah, and *opdP*-H11::ISphoA/hah between log FC −2 and −3 (Fig. 6B). Isolate 1499564 also had depletions within a flagellar operon and a putative coproporphyrinogen oxidase operon (Fig. 6B). Isolate 1378361 (no detectable transcript of *oprD* or *opdP*) and *oprD*-E12::ISphoA/hah were the only two isolates that showed depletions in genes encoding the chaperon proteins DnaK and GroEL (Fig. 6B). RT-qPCR was used to validate the differential expression observed across isolates (Table 3). The trends were similar; however, a few discrepancies were observed. Isolate 1203673 had few DEGs that reached the significance cut-off in the RNAseq data; however, RT-qPCR showed these operons followed the same trends as the other strains, with nitric oxide reductase and nitric reductase operons depleted. In all isolates, *creD* enrichment in the RNAseq data were greater than the increase in transcript levels seen in RT-qPCR (Table 3). Many of the DEGs within all five isolates were not annotated or were indicated as hypothetical proteins, which poses a challenge for analysis on the changes under imipenem challenge. All DEGs observed for these isolates can be found in File S2.

## DISCUSSION

Relebactam was designed to be paired with imipenem as an inhibitor for β-lactamases including the chromosomal AmpC of *P. aeruginosa*. In many cases, *P. aeruginosa* isolates resistant to imipenem alone have decreased and often susceptible imipenem/relebactam MICs. However, the major mechanism of resistance to imipenem is the downregulation of the porin, OprD (23, 24, 26). How then, does relebactam help to lower the imipenem MICs when decreased production of the porin OprD is the mechanism attributed to imipenem resistance? It has typically been recognized that overproduction of AmpC does not play a role in imipenem resistance unless modifications in the hydrolytic properties of AmpC accompany the overexpression of the enzyme (15). Modifications associated with *bla_ampC_* alleles (PDCs), especially T105A, are capable of imipenem hydrolysis (6) when AmpC is overexpressed. However, there was no pattern associated with imipenem resistance with the *bla_ampC_* alleles (PDCs) evaluated in this study (Table 1) which is supported by Young et al. and Delgado-Valverde et al. (9, 20). The data in this study strongly suggested that the *bla_ampC_* alleles present in these isolates did not contribute to the imipenem non-susceptible MICs in isolates with low basal transcript and is supported by the findings of Berrazeg et al. (7). The data presented in this study also suggested these *bla_ampC_* alleles have minimal impact on imipenem/relebactam MICs. Isolate 1378361 encodes *bla_ampC_* allele PDC-1 with no detectable *oprD* transcript and was susceptible to imipenem/relebactam. Conversely, isolates 1219235 and 1217799 have PDC-3 and PDC-8, respectively, and both have premature stop codons within *oprD*, yet these isolates were also susceptible to imipenem/relebactam. The two stable derepressed mutants in this study (1214215 and 763834) had the T105A mutation and were non-susceptible to imipenem/relebactam. Isolate 1214215 encoded PDC-3 and encoded 68 amino acid substitutions in OprD. Isolate 763834 encoded PDC-24 having 25 amino acid substitutions and in addition two amino acid deletions in OprD. Modifications in the amino acid sequences and transcript levels between *bla_ampC_* alleles and OprD may represent interplay between these two mechanisms resulting in variability in imipenem MICs. Although *oprD* RNA may be detectable, amino acid substitutions may decrease the efficiency of imipenem entry. Most of the OprD protein sequences had premature stop codons and were truncated (Table 2). The remaining isolates harbored multiple mutations that may lead to misfolding and premature degradation (25 or more residue changes) or may be responsible for reduced uptake efficiency (10 or fewer residue changes). However, the *bla_ampC_* alleles alone did not seem to have a major impact on imipenem susceptibility. Imipenem non-susceptibility in the isolates evaluated in this study was attributed to the lack of OprD. However, these data did not provide an explanation as to how relebactam lowered imipenem MICs in 64% of clinical isolates tested and in the OprD transposon and CRISPR mutants (Table 1).

In the presence of imipenem all the clinical isolates were found to have increased *bla_ampC_* transcript and produce the AmpC protein (Fig. 3). All but five of these isolates showed a decrease in imipenem MICs in the presence of relebactam (Table 1). These data suggest that AmpC plays a role in imipenem susceptibility when the enzyme is overproduced if the basal level of *bla_ampC_* transcript is low. However, the induction potential of the individual clinical isolates was not the same. Two isolates, 1214215 and 763834, were derepressed mutants as the basal level of RNA was 26- and 296-fold higher than PAO1 and ≥3-fold higher than the other clinical isolates (Fig. 1). The imipenem MIC decreased in isolate 763834 when relebactam was introduced but remained above the resistant breakpoint for both derepressed mutants (Table 1). The level of induction observed in an isolate is directly related to the basal level of *bla_ampC_* transcript and could play a role in the ability of relebactam to inhibit AmpC. Although differences were observed in the induction potential of these isolates, mutations in genes involved in the induction pathway were not identified that could account for the variation observed in AmpC induction (Table 2).

Variations in AmpC induction could also be a result of entry into the cell by less efficient porins such as OpdP. Recent literature has indicated that the outer membrane porin OpdP may allow entry of imipenem (18, 19). It is interesting to note that while whole-genome sequence analysis revealed a myriad of mutations within *oprD* sequences compared to PAO1, very few alterations were observed within *opdP*. Interestingly, an isolate (1378361) was observed that did not have detectable RNA amplification of *oprD* or *opdP* and OprD β-barrel structural changes were observed using structural modeling (data not shown), but the OpdP amino acid sequence was identical to PAO1. Despite lacking detectable porins OprD and OpdP, *bla_ampC_* induction occurred in the presence of imipenem. However, in the absence of a double knock-out of OprD and OpdP, the total contribution of OpdP cannot be ascertained.

Transcriptomic analysis of isolates treated with imipenem indicated a decrease in several operons related to nitrite metabolism, nitric and nitrous oxide metabolism, flagellar proteins, and several heat shock protein-related chaperon proteins (Fig. 6B). These data suggested *P. aeruginosa* undergoes a shift away from metabolic and motile processes when encountering a compound that compromises peptidoglycan integrity (imipenem). As expected, *creD* and *bla_ampC_* were both enriched under imipenem challenge. CreD has been reported to act as a global virulence regulator and play a role in AmpC regulation (34). Unfortunately, many genes that were differentially regulated were not annotated or denoted as hypothetical proteins. There are no studies investigating the transcriptomic modifications of *P. aeruginosa* in the presence of imipenem. However, a single study measuring the transcriptomic response to imipenem in *P. putida* showed differential regulation of genes encoding redox-related proteins and heat-shock proteins (35). However, *bla_ampC_* was not upregulated, highlighting the significant differences between these two species.

The data presented in this study showed that imipenem could induce AmpC and enter the cell in the absence of OprD providing a rationale behind the reduction in imipenem MICs when used in combination with relebactam. Many transcriptomic modifications occurred in the presence of imipenem and were dependent on the clinical isolate, suggesting differences in the selective pressure from which the isolate was collected. We have also identified operon differential expression including nitrite reductase, nitric oxide reductase, and DnaJ/K among diverse isolates indicating a phenomenon worth further investigation.

## MATERIALS AND METHODS

### Bacterial strains and growth conditions

A list of the strains used in this project is given in Table 1. *P. aeruginosa* PAO1-derived knock-out mutants were obtained from the Manoil Lab transposon mutant library (36) and were confirmed via PCR for the presence of the transposon and the absence of the associated gene. RT-qPCR was performed for genes located near the disrupted targets to ensure no polar effect from the transposon insertion (Table S1). Creative Biogene constructed a CRISPR KO of *oprD*, OprDKOCR. The KO was used to validate the results of phenotypic and genotypic experiments conducted with *oprD*-E12::ISphoA/hah (Table 1). OprDKOCR was then whole-genome sequenced by us to validate the complete deletion of the gene. The cells were plated from frozen 10% glycerol stocks upon TSA with 5% Sheep Blood and grown overnight at 37°C. Isolates were inoculated in 95 mL of cation-adjusted Mueller-Hinton broth at an optical density of 0.1 (Spectra, OD_600_). Isolates were grown in a shaking incubator at 37°C to mid-logarithmic phase, denoted by OD of 0.5. Plating and counting following serial dilution from mid-logarithmic cultures showed these populations to be 1 × 10^10^ cells across each strain.

### Antibiotic susceptibility testing

The MICs for PAO1, *oprD*-E12::ISphoA/hah, and *opdP*-H11::ISphoA/hah were determined using gradient diffusion using BioMérieux Etest for all except imipenem/relebactam (Liofilchem) and interpreted by CLSI guidelines (37). The *P. aeruginosa* strain ATCC 27853 was used as a quality control for drug potency and testing accuracy. The MICs for the clinical isolates were determined using broth microdilution and performed by International Health Management Associates, Inc (IHMA). These were validated in our lab using gradient diffusion. Rifampicin disk diffusion test was performed by CLSI guidelines with the *Escherichia coli* strain ATCC 29522 used as a quality control for drug potency (37).

### RT-qPCR

Isolates were grown according to method listed above and harvested at the mid-logarithmic phase. RNA extraction was carried out using TRIzol Reagent (Invitrogen, ThermoScientific). TURBO DNase (Invitrogen, ThermoScientific) was used to remove DNA contaminants and the RNA was quantified on an Eon spectrophotometer (BioTek). All RNA extracts were visualized on 1% agarose gel before and after DNase treatment to check for integrity and DNA contamination. For the imipenem induction experiments, after growth to an OD_600_ of 0.5, cultures were treated with 1/4th the respective MIC concentration of imipenem and grown for 15 min prior to total RNA isolation. RT-qPCR was performed using QuantiNova SYBR Green 1 Step kit (Qiagen) with 40 cycles of amplification at 57°C performed on an ABI 7500 (Applied Biosystems). Fold-change in transcript levels was determined using the 2^–∆∆Ct^ method (38) using *sodB* as an endogenous control and a no reverse transcriptase sample was used to check for DNA contamination. Coefficient of variance was calculated for the Cts for each gene target and found to be <9%. All experiments were performed in triplicate with independent growth and collection. All primers used for RT-qPCR experiments are located in File S3.

### Immunoblot

Custom polyclonal antibodies specific for AmpC and OprD were generated by GenScript. The epitopes for each are listed in File S3. The linear range of detection for the anti-AmpC and anti-OprD antibodies as well as Stain-Free fluorescence was determined via a dilution series western blot using total protein concentrations ranging from 2.5 to 45 µg (Fig. S2 and S3). The AmpC and OprD antibodies were diluted 1:20,000 and 1:30,000, respectively, for immunoblot for all analyses.

Isolates were grown according to method listed above and harvested at the mid-logarithmic phase. Whole-cell lysates were collected by bead-beating (Next Advance Bullet Blender) and protein concentrations were determined using a Bicinchoninic Acid Assay (Pierce BCA Protein Assay, ThermoScientific) on an Eon spectrophotometer (BioTek). Total protein lysates were separated via SDS-PAGE and normalized across the isolates using Stain-Free (Bio-Rad) fluorescent signal intensity. Following overnight primary antibody incubation, the secondary antibody was used with a dilution factor of 1:40,000. To ensure antibody specificity to OprD protein, *oprD*-E12::ISphoA/hah was used as a negative control (Fig. S1). OprD levels were determined and compared to PAO1 via densitometry using Image Lab (Bio-Rad) and Microsoft Excel. All protein experiments were performed in triplicate with independent growth and collection.

### Whole-genome and transcriptomic sequencing

Following growth, DNA from isolates was extracted using a MagAttract Microbial DNA Kit (Qiagen) and whole-genome sequencing was performed either on an Illumina MiSeq or completed by SeqCenter using combination of Illumina and Nanopore sequencing. Following RNA isolation, all samples were sent to SeqCenter for transcriptomic sequencing and analysis, using Illumina Ribo-Zero Plus rRNA Depletion and Illumina sequencing. Reads were quality checked and adapters trimmed using bcl-convert (39) and mapped using HISAT2 (40). At least 96% of base pairs scored above Q30 among all isolates. Total mapped RNA reads among samples were ~3 × 10^7^ and rRNA reads were depleted well below 1 × 10^6^(File S4). Reads were quantified using Subread’s featureCounts (41) and the counts normalized through edgeR’s (42) Trimmed Mean of *M* values algorithm. Normalized counts were converted to counts per million and differential expression analysis was undergone using edgeR’s Quasi-Linear *F*-test functionality against treatment groups. For comparative total transcriptomic analysis, RNA transcripts from each untreated isolate were compared to RNA transcripts from untreated PAO1. For the imipenem induction analyses, RNA transcripts from the imipenem-treated isolate were compared to RNA transcripts from the same untreated isolate. Genes were determined to be significantly differentially expressed with a │logFC│> 1 and *P* value < 0.05. All RNAseq analyses were performed in triplicate with independent growth and collection.

## Data Availability

Whole-genome assemblies uploaded to GenBank NIH sequence database under BioProject ID PRJNA1113550. Raw Illumina RNA read data uploaded to the Sequence Read Archive (SRA) NIH database under BioProject ID PRJNA1113633.

## SUPPLEMENTAL MATERIAL

Figure S1 spectrum.00142-24-s0001.tif OprD protein.

Figure S2 spectrum.00142-24-s0002.tif AmpC protein linearity curve.

Figure S3 spectrum.00142-24-s0003.tif OprD protein linearity Curve.

Figure S4 spectrum.00142-24-s0004.tif Rifampicin disk diffusion test.

Supplemental File S1 to S4 spectrum.00142-24-s0005.xlsx RNAseq Data and primers.

Table S1 spectrum.00142-24-s0006.docx Transcript levels of genes down stream of transposon inactivated genes.

ASM does not own the copyrights to Supplemental Material that may be linked to, or accessed through, an article. The authors have granted ASM a non-exclusive, world-wide license to publish the Supplemental Material files. Please contact the corresponding author directly for reuse.
