# Supplementary figures and images for: AmpC induction by imipenem in Pseudomonas aeruginosa occurs in the absence of OprD and impacts imipenem/relebactam susceptibility
Source: Microbiol Spectr. 2024 Sep 24;12(11):e00142-24. doi: 10.1128/spectrum.00142-24 (PMC11537110; doi:10.1128/spectrum.00142-24)

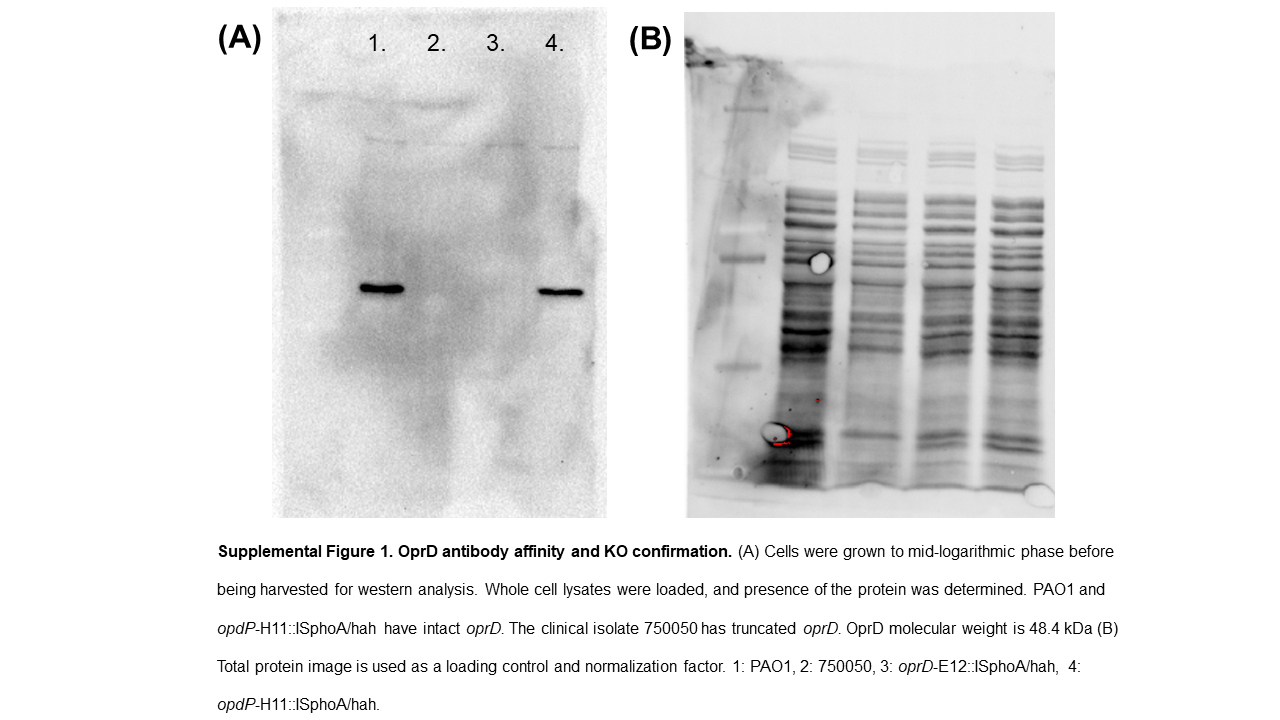

Supplement: Figure S1 — OprD protein. [file spectrum.00142-24-s0001.tif]

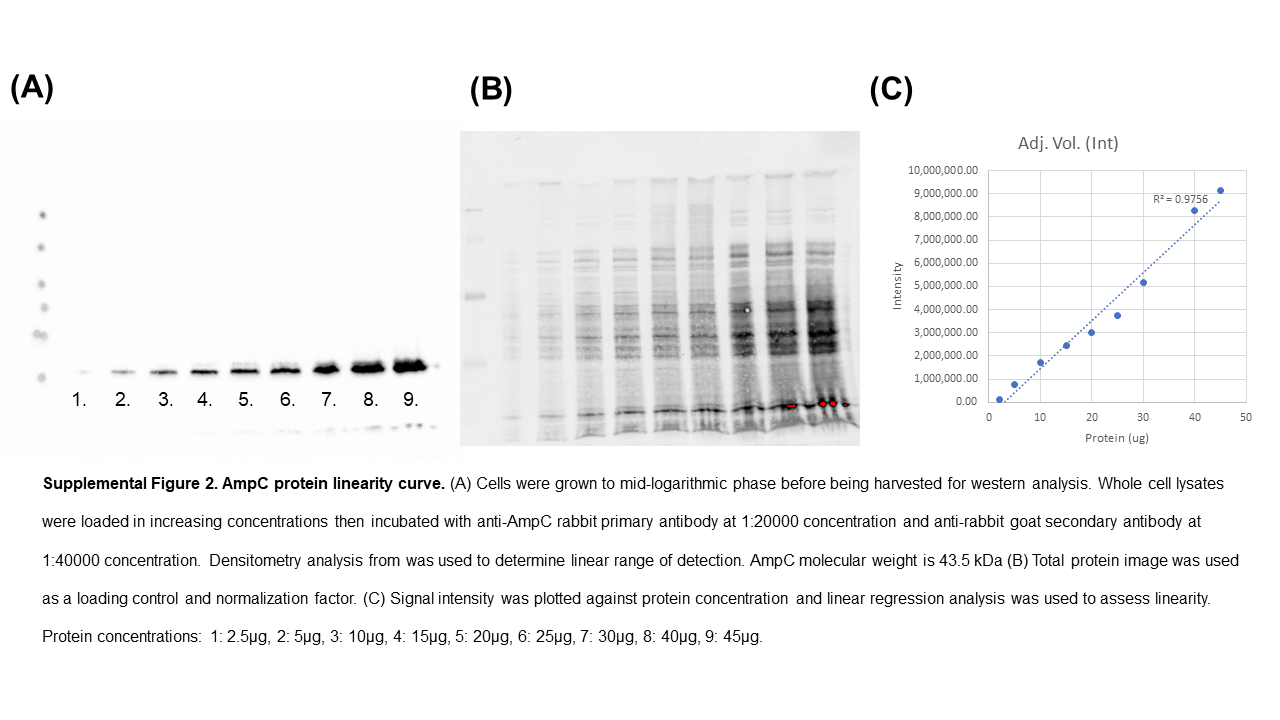

Supplement: Figure S2 — AmpC protein linearity curve. [file spectrum.00142-24-s0002.tif]

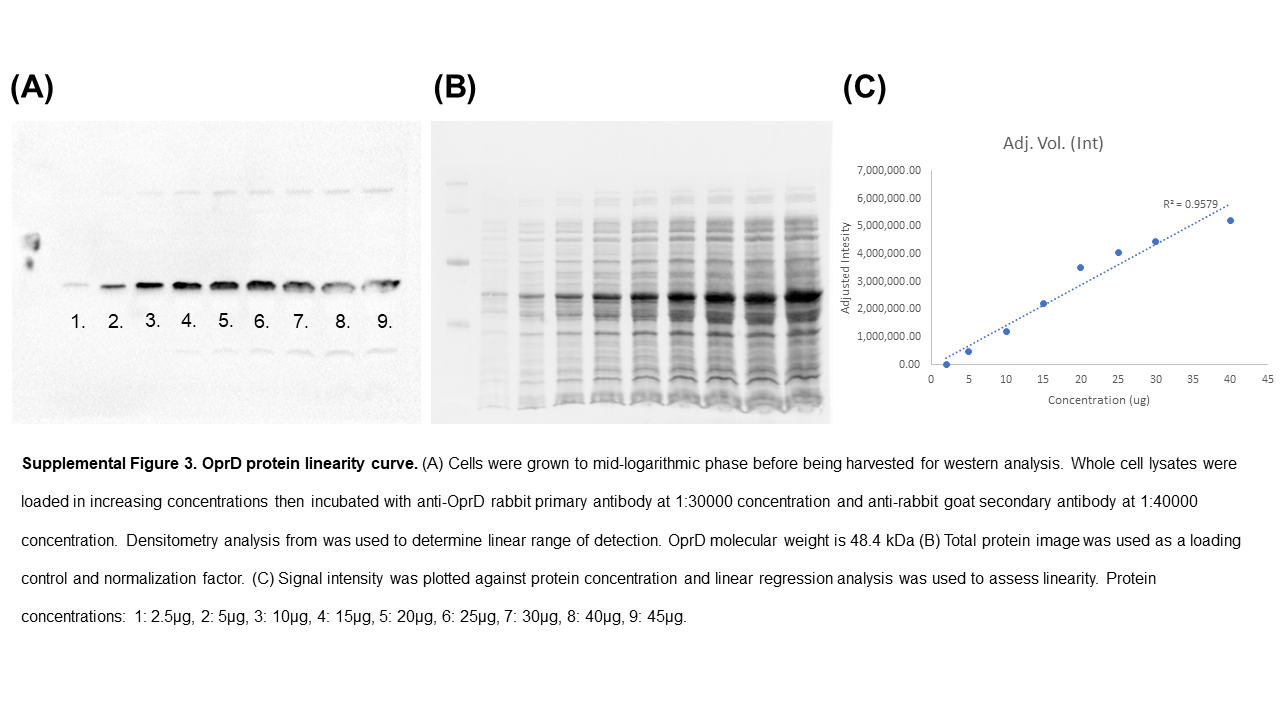

Supplement: Figure S3 — OprD protein linearity Curve. [file spectrum.00142-24-s0003.tif]

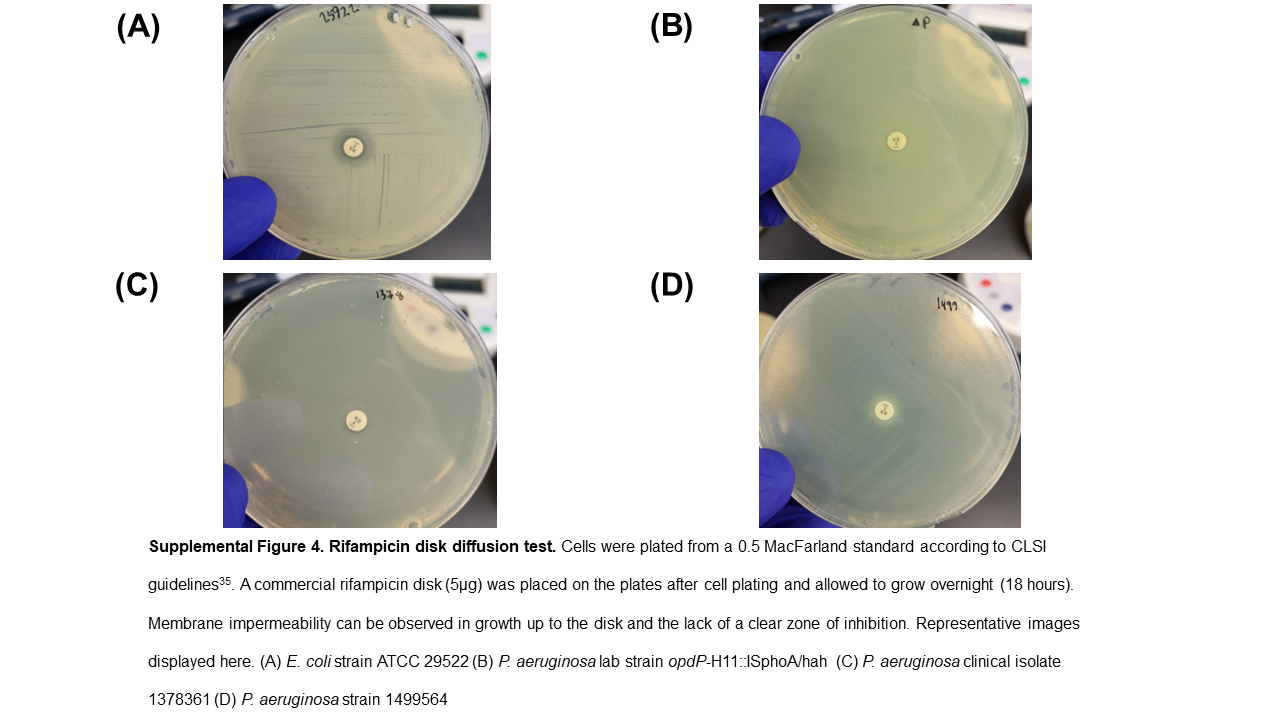

Supplement: Figure S4 — Rifampicin disk diffusion test. [file spectrum.00142-24-s0004.tif]
