# Supplementary material for: AmpC induction by imipenem in Pseudomonas aeruginosa occurs in the absence of OprD and impacts imipenem/relebactam susceptibility
Source: Microbiol Spectr. 2024 Sep 24;12(11):e00142-24. doi: 10.1128/spectrum.00142-24 (PMC11537110; doi:10.1128/spectrum.00142-24)
Supplement: Table S1 — Transcript levels of genes down stream of transposon inactivated genes. [file spectrum.00142-24-s0006.docx]

| **OprD** | | **OpdP** | |
| --- | --- | --- | --- |
| **pa0959** | **dps** | **dppb** | **dppa4** |
| -2 | -1 | 1 | 1 |

**Supplemental Table 1. Transcript levels of genes down stream of transposon inactivated genes.** Fold-change in transcript levels was determined comparing transposon insertion knock-out strains to the parent PAO1 strain. RT-qPCR analysis conducted on two genes each located downstream of the respected gene disruption. Fold-change was calculated using 2^-ΔΔCT^ method.
